# Plasma-Activated Water (PAW) as a Disinfection Technology for Bacterial Inactivation with a Focus on Fruit and Vegetables

**DOI:** 10.3390/foods10010166

**Published:** 2021-01-15

**Authors:** Aswathi Soni, Jonghyun Choi, Gale Brightwell

**Affiliations:** 1 Food Assurance, AgResearch, Palmerston North 4442, New Zealand; Gale.Brightwell@agresearch.co.nz; 2 The New Zealand Institute for Plant and Food Research Ltd., Private Bag 3230, Waikato Mail Centre, Hamilton 3240, New Zealand; Jonghyun.Choi@plantandfood.co.nz; 3 New Zealand Food Safety Science Research Centre, Palmerston North 4474, New Zealand

**Keywords:** cold atmospheric plasma, microbes, disinfection, non-hazardous, inactivation, foodborne pathogen

## Abstract

Plasma-activated water (PAW) is generated by treating water with cold atmospheric plasma (CAP) using controllable parameters, such as plasma-forming voltage, carrier gas, temperature, pulses, or frequency as required. PAW is reported to have lower pH, higher conductivity, and higher oxygen reduction potential when compared with untreated water due to the presence of reactive species. PAW has received significant attention from researchers over the last decade due to its non-thermal and non-toxic mode of action especially for bacterial inactivation. The objective of the current review is to develop a summary of the effect of PAW on bacterial strains in foods as well as model systems such as buffers, with a specific focus on fruit and vegetables. The review elaborated the properties of PAW, the effect of various treatment parameters on its efficiency in bacterial inactivation along with its usage as a standalone technology as well as a hurdle approach with mild thermal treatments. A section highlighting different models that can be employed to generate PAW alongside a direct comparison of the PAW characteristics on the inactivation potential and the existing research gaps are also included. The mechanism of action of PAW on the bacterial cells and any reported effects on the sensory qualities and shelf life of food has been evaluated. Based on the literature, it can be concluded that PAW offers a significant potential as a non-chemical and non-thermal intervention for bacterial inactivation, especially on food. However, the applicability and usage of PAW depend on the effect of environmental and bacterial strain-based conditions and cost-effectiveness.

## 1. Introduction

Food spoilage is defined as a change in any food product that leads to a significant reduction in its sensory qualities, such as color, texture, and overall smell, due to physical damage or chemical changes (e.g., oxidation), and thus rendering it unacceptable by the consumer [1]. These changes are mainly the result of microbial growth and metabolism in the food, which may lead to the production of enzymes that facilitate to reactions resulting in deleterious by-products affecting the food. These by-products vary in different types of food and can lead to adverse sensory properties, including the presence of slime, off-odors, and off-flavors. Bacterial strains associated with spoilage include *Pectobacterium carotovorum* [2,3], *Brochothrix thermosphacta* [4], *Clostridium perfringens* [5], *Bacillus spp.* [6,7], *Pseudomonas fragi* [8], *Pseudomonas fluorescens* [9], *Shewanella putrefaciens* [10], *Serratia liquefaciens* [11] and *Hafnia alvei* [12]. Food spoilage is a primary concern for food industries due to susceptible loss of shelf life and hence the economic losses followed by a long-term impact on consumer preferences. Nevertheless, food spoilage is also a threat to the environment as it leads to excessive wastage that end ups in the landfill, which does not contribute to sustainable living. This is supported by a survey conducted in 2018, which indicated that 30–50% of the total food produced exclusively by one country in a year ends up in the landfill, with the contribution from households, processing industries, food services, primary production sector and retails being 53%, 19%, 12%, 11%, 5%, respectively [13]. Minimizing food spoilage by employing multiple interventions might help not only the food industries but also the environment. 

Another concern for the food processing industries and the regulatory authorities is food poisoning due to bacterial growth in food. Some bacterial strains are capable of producing toxins under certain conditions either in the food itself or inside the human body once live bacterial cells are ingested, while others are enteropathogenic and entero-invasive pathogens [14]. Few examples of foodborne pathogens include *Clostridium botulinum* [15,16], *B. cereus* [17], *Staphylococcus aureus* [18,19], *Listeria monocytogenes* [20], *Salmonella enterica* serovar typhimurium [21], *Salmonella* spp. [22], *E. coli* O157:H7 [23], *C. perfringens* [24], *Shigella* spp. [25], *Yersina* spp. [26] and *Campylobacter jejuni* [27]. Food poisoning has been a major public health concern, particularly regarding outbreaks affecting immunocompromised individuals and infants and thus may lead to adverse social and economic effects. Although many food-poisoning cases go under-reported due to quick recovery and almost minimum effect on healthy individuals, some still have adverse effects on immunocompromised individuals and infants [28]. Alternate disinfection technologies that do not employ thermal treatments or harmful chemicals could be valuable options for minimizing contamination and growth of microbial contaminants leading to either spoilage or food poisoning. Such sustainable technologies are of great importance to the vast ever-growing population with increasing demand of food across the globe. 

Recently, the application of advanced oxidation processes (AOPs) for the decontamination of fruit and vegetables has been widely investigated. These technologies include electrolyzed water [29,30], gaseous ozone [3,31], UV light [32,33], and cold plasma [33,34]. One of these oxidation technologies is plasma-activated water (PAW), which is generated by treating water with cold atmospheric plasma (CAP) using controllable parameters such as plasma-forming voltage, carrier gas, temperature, pulses, or frequency as required. Plasma has been recognized as the fourth state of matter. It is the ionized gas usually produced when gas molecules are exposed to the electric field, forming reactive species and ions [35]. PAW has received significant attention from researchers over the last decade due to its non-thermal and non-toxic mode of action, which is mainly due to the reactive species that could react with the bacterial structural components and later organelles, leading to death [36]. This review summarizes the role of PAW as a disinfectant for bacterial decontamination on the surface of foods with a focus on vegetables and fruit [36,37,38,39,40]. Although most of the studies have investigated the combined effect of PAW with other interventions such as heat, their standalone effect has also been reported. 

## 2. Systems for PAW Generation

The fundamental method of generation of PAW involves operating a plasma generator inside the water to generate the ions, which lead to reactive species for bacterial inactivation. There are various combinations and models in the literature leading to difference in the final outputs; these are outlined in Table 1. 

Most studies have indicated an immediate drop in pH and an increase in electrical conductivity and the ORP as a result of the formation of reactive species in the PAW samples (Table 1). However, the increase of change in these properties cannot be directly correlated with a single factor or reason. When PAW is produced, the gaseous species from either the working or the atmospheric gas enters the liquid-gas interface and as a result there are complex reactions leading to the non-equilibrium, hence generation of the ionic moieties [44,47,48]. This process is highly influenced by the electric field and also using bubble implosions which hence the movement as well as dispersion of the phenomenon across the interface [49]. A recent review suggests that the electrical breakdown in water can occur without a phase change such as evaporating liquid and condensing or dissolving vapor [48]. The factors affecting the changes in PAW during activation may depend on multiple factors. For example, increase in discharge power, which is a direct function of applied voltage, would affect the increase in electric conductivity of the PAW [47]. On the other hand, in another study Vlad et al. showed that increase in treatment time would increase bacterial inactivation by PAW [50]. Although these studies reported above (Table 1) have used different set ups for the PAW generation, it could still be concluded that the efficiency can be a combined effect of two or more factors such as PAW activation time, temperature, power used and the aeration or bubbling to improve the formation of reactive oxygen species (ROS) [51].

### Physicochemical Properties of PAW 

PAW shows lower pH, higher conductivity and higher oxygen reduction potential when compared with untreated water [44,52]. The reduction in pH is due to the formation of acidic chemical species, which result in a steep decrease from pH 7 to pH 3 within 5–10 min of activation, but with little change thereafter [37,53]. Oxidation-reduction potential (ORP) can be defined as the ability of any solution to acquire or loose electrons to an electrode, and this property of PAW is much more prominent as compared with non-activated water. ORP of PAW depends on the strength of activation, which further depends on the applied voltage, carrier gas and other parameters leading to an increase of up to 63% [54]. Conductivity is the ability of any solution to allow current to pass through it and is reported to significantly increase due to plasma activation, primarily because of the generation of ions [45]. With a plasma jet that was operated from a 10 kHz sinusoidal high-voltage power source with 18 kV peak-to-peak AC voltage using pre-mixed oxygen and argon, the pH reduced from 7 to 3, ORP increased from 250 to 550 mV, conductivity rose from 0 to 410 μS/cm and temperature increased from 25 to 30 °C after 15 min of activation [55]. With a similar plasma source, when PAW was produced using 0.40–0.42 kV AC voltage, the conductivity increased from 5 to 20 mS/cm, the ORP value increased from 180 to 250 mV, pH decreased from 7.0 to 6.0, and the temperature increased from 20 to 40 °C [56]. Hence, the change in the reactive species of PAW are measurable as ORP and pH, and these changes directly show their effect on the potential to attack and disrupt the bacterial membranes during inactivation [40]. These effects are explained in detail in this review. 

## 3. The Effect of PAW on Bacterial Inactivation

### 3.1. The Effect of PAW on Vegetables and Fruit

PAW has been known to show inactivation in the model systems such as water and buffer, indicating potential to be used in liquid food matrix. However, different food matrices can have different effects on the microbial adhesion and resistance, which could alter their efficiency in several ways. Table 2 summarizes the reports on microbial inactivation specifically on surfaces of vegetables and fruit. In most cases described here, the use of PAW is as a washing intervention resulting in inactivation of up to 3 log_10_ CFU/mL of different strains (Table 2). 

The efficiency of PAW cannot be directly compared with that of heat treatment such as thermal pasteurization which can lead to at least 6 log_10_ CFU/mL of non-spore-forming bacteria depending on the food matrix. However, PAW treatment has been reported to be effective in increasing the shelf life of fresh food. 

PAW has also shown positive results in inhibiting the growth of bacteria, molds, and yeasts in fresh-cut apples in a study by Liu et al. [42]. In this study, fresh-cut apple slices were immersed in PAW generated at 8 kV for 5 min at room temperature and the treated slices were stored at 4 ± 1 °C and 90% relative humidity (RH) for 12 days. Aerobic bacteria count reduced by 1.05 log_10_ CFU/g on day 12 of refrigerated storage [42]. Additionally, on day 12, a reduction equivalent to 0.64, and 1.04 log_10_ CFU/g was observed in molds, and yeasts, respectively. PAW treated apples showed absence in total aerobic bacteria for the first two days as compared with the 3 log_10_ CFU on the control samples which were immersed in distilled water and stored under the same conditions as PAW treated slices. After 12 days, the total aerobic count reached up to 4–5 log_10_ CFU/mL in PAW treated slices which were still less than the untreated sample by 2 log_10_ CFU/mL [42]. Yeast and mold numbers were also found to be lesser by 0.5 and 1 log_10_ CFU/g in the PAW treated samples as compared with the control. The delayed onset of bacterial growth and hence increase in numbers in the PAW treated samples were postulated as a result of sub-lethal injury due to the reactive species. A similar effect on shelf life using PAW as a disinfecting agent was observed on strawberries, where PAW treated (10 kHz sinusoidal AC voltage source at 18 kV) strawberries showed less than 1 log_10_ CFU/g inactivation of *S. aureus*. Complete absence of any growth of hyphae of filamentous fungi after six days of storage at 20 ± 2 °C and 70 ± 5% RH indicated a potential of PAW to counter fungal spoilage [55]. PAW has been reported to not only reduce the total aerobic counts but also reduced the ability of growth by fungi and yeasts, which indicates that PAW has the ability to alter the microbial community of the food-based ecosystem, which needs further investigation [57]. Interestingly, fungal growth has been reported to be significantly reduced by PAW treatment on strawberries by Ma et al. [55]. The rough and uneven surfaces of strawberries act as physical barriers to protect microflora from ethanol and UV light inactivation. However, PAW was could access these areas, indicating a potential of PAW over other interventions. 

### 3.2. The Effect of PAW on Microbial Inactivation in Model Systems

Buffers, diluents and salt solutions are often used as the dispersion matrix in place of real food for microbial inactivation assays. This not only helps to minimize the effects intrinsic characteristics and composition on the results, but also provides opportunity to modify the intrinsic factors according to specific requirements. The studies specifically reported on inactivation of non-spore-forming bacterial cells in model systems using PAW are listed in Table 3.

Bacterial spores are resistant to thermal treatment (based on the decimal reduction time or D-value), several chemicals, and dehydration. In most of the cases, either moderate to high thermal treatment or a combined effect of multiple inactivation technologies need to be applied for spore inactivation in food [64]. PAW has been investigated as an alternative to thermal treatments or harmful chemicals against bacterial spores. For example, a study by Bai et al. [65] showed that up to 2 log_10_ CFU/mL reduction in the population of *B. cereus* spores could be achieved by a single treatment of PAW at 55 °C (input power of 650 W for 60 s). This study also indicated that the inactivation kinetics fitted the log-logistic model, which assumes there could be differences in the inactivation kinetics of any bacterial population. As per this model, the whole bacterial spore population can be divided into subpopulations. The first subpopulation has spores that are entirely resistant to the treatment. The second has the ones that can repair themselves post-treatment and the third are fully vulnerable to the treatment [66]. According to Bai et al. [65], the inactivation was enhanced by reducing the volume of activation from 100 to 50 mL and by lowering the spore inoculum from 10^7^ to 10^5^ CFU/mL. 

The effects of PAW on biofilms have also been explored. A study by Xu et al. [63] indicated that both the plasma inducement time and the plasma treatment time could affect the bacterial cells in their ability to generate biofilms. Apart from more than 5 log_10_ CFU/unit reduction of *S. aureus* cells in the biofilm, the regrowth capacity of the surviving cells was reduced by 30% as compared with the untreated cells [63]. This effect is postulated as a result of regulation of genes (*SarA*, *IcaA*, *SigB*, *Rbf*, *LuxS*) involved in biofilm formation, especially in response to disinfectants such as hydrogen peroxide. [63,67]. On the contrary, in a study by Charoux et al. it was seen that PAW as a standalone method was ineffective in inactivating *Escherichia coli* K12 cells in biofilms; however the synergistic effects of airborne acoustic ultrasound and PAW enhanced the inactivation potential by 2 log CFU/mL [68]. A study by Hozak et al. also indicated that PAW was ineffective in inactivation of Gram-positive and Gram-negative cells in the biofilm [69]. Similar results were obtained when PAW was found to be more efficient in inactivating *S. epidermidis*, *L. mesenteroides*, and *H. alvei* in planktonic forms as compared with adherent forms in biofilms [62]. Postulated reasons for the reduced effect by PAW against the bacterial cells in biofilm could be due to the inability to overcome the resistance of the bacterial cells in stationary phase unlike those from overnight culture (planktonic cells) in exponential phase. Alternatively, the short-lived reactive species, which are effective against the planktonic cells might not be effective in the dense consortium of the biofilm, which consists of extra cellular matric as well as well-organized clumps of bacterial cells. Although the mechanism of resistance of bacterial biofilms is yet to be investigated in detail, it can be concluded that PAW would need to be combined with at least one more technology to enhance its efficiency in cleaning regimes for biofilm removal. 

### 3.3. The Effect of PAW as a Hurdle Intervention against Bacterial Inactivation

PAW has also been investigated as a hurdle technology or a combined synergistic approach against bacterial inactivation, especially in case of bacterial spores. The combination of PAW at 40 °C and 55 °C for the decontamination of *B. cereus* spores resulted in an inactivation equivalent to 1.54 and 2.12 log_10_ CFU/g of *B. cereus* spores, respectively after 60 min of exposure [70]. The detailed analysis on the structure of the treated *B. cereus* spores using transmission electron microscopy, scanning electron microscopy and the propidium iodide (PI) assays indicated visible disruption of external structure (spore coat and cortex) along with leakage of intracellular contents [70]. PI is a fluorochrome capable of binding and labelling DNA fragments, only when there is some level of damage to an otherwise intact cell membrane, which leads to permeability of the PI into the cell [71]. 

*B. cereus* spores consist of multi-layered protective structure including a spore coat and a peptidoglycan cortex [72], which acts as a barrier to the external chemical disinfectants, including phenols, organic acids, quaternary ammonium compounds (QACs) biguanides and alcohols [73,74]. Images of spores treated with PAW showed specific disruption or damage on the external layers, which include the spore coat made of peptidoglycan layers. The structural damage is indicative of oxidative stress and mild thermal stress [70]. Ultrasound technology (40 Hz, 220 W) when combined with PAW at 40 °C for 60 min has been tested against *S. aureus* and *E. coli*. Ultrasound technology employs mechanical sound waves of low to high frequencies (20 kHz–1.5 MHz) to have multiple applications including bacterial inactivation, by imparting a damage to the cell walls as the mechanism of action [75]. In combination with PAW, this mechanism against cell structural integrity could be enhanced. As a result, inactivation equivalent to 1.33 log_10_ CFU/mL of *E. coli* K12 and 0.83 log_10_ CFU/mL of *S. aureus* was achieved, as compared with less than 0.5 log_10_ CFU/mL for both strains under the same conditions with PAW alone [36]. There was no significant difference among the pH, EC, and ORP [36] between the PAW samples treated with and without ultrasonication, indicating that any change in the parameters of acidity, EC and ORP was due to the presence of reactive species generated by plasma activation and not by ultrasonication. The PAW treatment used in this study was for a set time (up to 60 min) while being held at three different temperatures (4, 25 and 40 °C), followed by ultrasonication at 40 Hz and output power of 220 W. This study demonstrated that ultrasound increased the penetration of the reactive species generated by PAW into the bacterial cells, and though the inactivation was significantly different by <1 log_10_ CFU/mL when PAW treatment with and without ultrasound was compared, the scanning electron microscopic images showed increased porosity of the PAW and ultrasound treated samples as compared with the samples treated with de-ionized water [36]. PAW and mild heat (50 °C) for 6 min has been reported to be effective against *S. cerevisiae* leading to an inactivation equivalent to 4.4 log_10_ CFU/mL as compared with a relatively lower inactivation equivalent to 0.27 and 1.92 log_10_ CFU/mL with PAW at 25 °C and mild heat at 50 °C for 6 min as standalone treatments [76]. This study further investigated the damage caused on the cells due to treatment using scanning electron microscopy, which revealed the appearance of visible deformation on cell surface along with complete distortion of parts of the cell wall in contrast to the images showing cell integrity post-treatment by PAW alone and mild heat alone. The PI permeability assay also supported these findings where the intensity of penetration of dye increased significantly (*p* < 0.05, by 132.14-fold), indicating compromised cytoplasmic membranes and thereby increased binding of the dye to cellular DNA and RNA [76,77]. PAW and mild heat (50, 52.5, and 55 °C, 30 min) has been reported to inactivate *Saccharomyces cerevisiae* by 2.4 log_10_ CFU/g when inoculated on grapes [78]. *S. cerevisiae* has been reported in spoilage of fresh fruit and fruit juices [79]. Therefore, these studies on successful inactivation of yeast indicate a potential application of PAW towards extension of shelf life with minimum effect on the quality attributes.

In another study, the combined effect of PAW (18 kV for 120 min) and mild heat at 60 °C led to inactivation equivalent to 3.4 and 3.7 log_10_ CFU/g of *L. monocytogenes* and *S. aureus*, respectively, on salted Chinese cabbages [38]. The treatment was performed in a sequential approach where initial PAW treatment was performed for 10 min, followed by heating in a water bath (60 °C) for 5 min. Interestingly, this treatment of PAW (18 kV for 120 min) and mild heat at 60 °C also resulted in 4, 5.7, 4.0, and 2.6 log_10_ CFU/mL reduction in mesophilic aerobic counts, lactic acid bacteria, yeast, and molds and coliforms, respectively, which were all present in the background microflora of the untreated samples [38]. This study indicated a potential application of PAW with mild heat as a disinfection approach in Korean market for cabbage kimchi [38] which is preferably cleaned using a non-thermal approach to minimize changes in the sensory attributes for a fresh-like quality. Considering the studies reported, it can be concluded that PAW has demonstrated efficiency as a hurdle approach either in combination or sequentially to eliminate the bacteria or yeast from food surfaces. However, the impact on the formation of the reactive species in the food needs further research as a case-by-case basis. 

### 3.4. PAW-Mechanism of Action on Bacterial Cells

The mechanism of disinfection using PAW can be explained as the oxidative stress on the cell membranes of the bacterial cells. The cell membrane is disrupted, followed by further damages to organelles, proteins and nucleic acid leading to the cell death [45,61]. The inactivation of bacterial cells by PAW has been reported as an effect of the reactive species on the bacterial cell membranes and the organelles. The reactive species can interact with and hence damage the bacterial cell membranes through lipid peroxidation, which disrupts the structure followed by leakage, morphology change, DNA damage and disruptions of the functional structures of the proteins [80]. There is a clear agreement in the literature on the dominant reactive species responsible for the mechanism of action against bacterial cells. For example, hydroxyl radicals, ozone, nitric radicals (NO_2_^−^), and nitrate ions (NO_3_^−^) are considered capable of interacting with the biological components of the cell membrane and later the cytoplasmic components and organelles inside the bacterial cell. The process of structural disruption leads to functional damage and physiological imbalance. For example, three reactive species (NO_3_^−^, H_2_O_2_, O_3_) generated in PAW using a dielectric barrier discharge (DBD)-atmospheric cold plasma (ACP) system showed an effect in inhibiting the growth of spoilage microbes as measured through total viable counts on shrimps [81]. In this case, the total microbial numbers increased significantly (*p* < 0.05) from 3.9 to 8.6 log_10_ CFU/g during nine days of storage in control samples that were washed using tap water, whereas the growth of microbes were slower when washed with PAW. The increase was only from 3.9 to 4.4 log_10_ CFU/g for the initial 6 days, and reached 6.5 log10 CFU/g on the 9th day when stored at 5 °C, indicating possible sub-lethal stress on the initial population, which might have hindered the growth rate [81]. This study did not establish the individual effect of each of the reactive species. However, in general, reactive species are known to attack intracellular superoxide dismutase (SOD), catalase (CAT), peroxidase, and oxidases, which impart the form the defense mechanism of the bacterial cells against stress [82,83]. This initial resistance is overcome when the concentration of reactive species increases above the capacity of in-built defense systems, leading to commencement of the oxidative damage involving lipids, DNA, and proteins. This process is defined as oxidative stress, which can lead to cell death [84,85]. Short-lived reactive species such as oxygen radicals lead to the immediate lethal effect on the bacterial cells, whereas long-lived species such as nitrate ions, hydrogen peroxide radicals and ozone might lead to a reduction in the recovery and growth of the sub-lethally injured cells [81]. The reduction in pH was only a difference of 0.2 units and in this case, the reduction in microbial growth rate cannot be attributed to decrease in pH balance outside and hence inside the cells [81]. Additionally, the gas used for the creation of the PAW does not seem to indicate a direct influence on the inactivation of bacterial cells [81]. However, this needs further investigation before being established as a fact. 

## 4. The Effect of PAW on Sensory Attributes and Consumer Acceptance

Consumer demand for fresh-like, minimally processed or natural food has been a growing area of interest for food scientists and industries. These trends call for technologies that only result in insignificant or very mild changes due to physical, and chemical reactions that affect the “fresh-like quality” [86,87,88]. PAW is a chemical-free and non-thermal intervention and has the potential towards achieving the minimal processing target. However, there are no sensory studies using trained panels reported in the literature. The changes associated with quality and appearance, which also contribute to likability, have only been investigated at the laboratory scale. For example, Chinese bayberries treated by PAW showed a significantly (*p* < 0.05) higher firmness (measured using a texture analyzer) by 1.2 newton (N) when stored at 3 °C for eight days as compared with untreated berries [89]. The PAW treatment used in this study was a hollow electrode dielectric barrier structure (HEDBS) with air as the working gas at a flow rate of 260 L/h using an AC power source with a frequency of 20 kHz. The treatment time in this study was 0.5 min at room temperature (25 °C) [89]. In red grapes, samples treated with PAW had a higher rate of color change (red or violet to dark violet) than untreated controls [89], as assessed using three parameters of color index for red grapes (CIRG), namely lightness (L), hue angle (H) and chroma (C), over eight days of storage at 3 °C [90]. The change in color was attributed to the impact of ROS on anthocyanins [89]. However, in response to this oxidative stress, these berries were found to produce more antioxidants and accelerate the antioxidant enzymes to scavenge excessive ROS, which also related to the higher CIRG value as compared to the control [89]. 

In a similar study, the effect of PAW (11 kV for 20 min) on the color (L, a, and b values) of spinach leaves after storage at 4 °C for eight days was compared with the changes in the untreated samples [59]. The control (untreated) and PAW treated samples showed no significant difference in color evaluated using L, a, and b values, which indicated the “fresh-like appearance”. It was shown that multiple rinsing with water affected the color as there was an increase in the yellowness (b values), which was not seen with the PAW treated samples [59]. In another study by Ma et al. [55] using PAW treatment (10 kHz sinusoidal high-voltage source at 18 kV), the change in color and firmness of the treated and untreated strawberries were compared after storage at 20 ± 2 °C and 70 ± 5% RH for four days [55]. No significant difference in color (L, a, and b values) and firmness (N) was observed between the treated and untreated strawberries, which showed that PAW did not induce any change in these attributes and hence would resemble fresh-like qualities. However, the firmness of the treated and the untreated samples was reduced after storage when compared with the results obtained on day zero, indicating a natural phenomenon of loss of the turgor pressure post-harvesting, especially when held at temperatures above refrigeration [55]. Color is considered to be a very influential parameter of consumer’s perception of food’s healthiness and purchase intention. Hence, the reports on the color changes are indicative of consumer preference and perception [91]. The absence of any significant change on color would indicate minimum effect of PAW on fruit and vegetables and can be perceived as “minimally processed”.

## 5. Future Implications, Research Gap, and Conclusions

PAW has promising potentials to be used as a non-chemical decontamination agent for food. Although no harmful chemicals are used in the generation of PAW, the presence of residual reactive species in food during the shelf life is an area of investigation that remains underexplored. At the same time, the cost and energy required to generate efficiency against bacterial contaminants are high. The research gap on the specific effect of microbial inactivation and the impact of specific intrinsic and extrinsic food properties opens opportunities for comprehensive research and application. Therefore, PAW could emerge as a non-hazardous, non-chemical disinfectant for food industries which could be used as a hurdle or as a standalone step depending on the microbial population being targeted. The successful and safe application of PAW in food-grade systems would also depend on the optimization of analytical methods for detecting reactive species with minimum interference due to the composition of food being tested.

Most of the work done on PAW as a disinfectant is on food (fruit and vegetables) surface in lab settings where the controlled environment helps to identify variables and mechanism of action. There is a significant gap in the literature on the use of PAW as a disinfectant in real-life supply chain scenarios (e.g., packaging house and cool store). At the same time, the specific compositional changes due to any residual reactive species needs to be studied throughout shelf life to ensure that the structural changes of the food (if any) do not affect any sensory attributes. The effect of PAW on bacterial spores food and model systems need an elaborative investigation. Also, it is important to confirm that the reactive species in PAW do not lead to the germination onset in spores as this could adversely affect the shelf life or safety of food. Alternatively, if PAW can induce germination, it has the potential to be used as a hurdle before thermal inactivation thereby reducing the heat exposure and energy required to inactivate bacterial spores. There is no evidence in the literature to compare the effect of PAW of the decimal reduction time (D-value) of spores and vegetative bacterial cells, highlighting the research gap on whether this resistance (D-value) is comparable to the well-established thermal resistance, which is much higher in spores. Similarly, there is a lack of direct comparison of the resistance exhibited by Gram-positive and Gram-negative vegetative cells. Future work in these areas would enhance the knowledge of PAW disinfection in food processing.

## Figures and Tables

**Table 1:** The effect of different generation conditions and the characteristics of PAW.

Gas and Additional Features	Gap (Between Water Surface and the Upper Electrode)	AC Voltage and Frequency	Quantitative Changes after Generation Time	References
Grounded copper electrode (diameter 0.5 mM) on top and a capillary tube to generate bubbles	10 mM	3–6 kV, 3–10 kHz	pH changed from 6.75 to 3.77 and NO_2_^−^ concentration changed from 3.77 μM to 8.686 μM in 15 min of activation	[41]
Plasma jet unit coaxial tungsten electrode and a quartz tube (diameter of 700 μM)	nil	6–10 kV, 7.0 kHz	Not determined	[42]
Plasma jet with RD1004 rotating nozzle	8.1-cM	voltage (295 V), air pressure (1990 mBar), and frequency (22.5 kHz)	pH changed from 6.5 to 3.1 and Oxidation-reduction potential (ORP) increased from 376.54 to 534.52 RmV in 5 min	[43]
Atmospheric pressure plasma jet (patent atmospheric pressure plasma jet (APPJ))	0 mM	3.0 kV and 16 kHz	pH reduced from 7 to 3.2 in 20 min and the ORP increased from 310 to 510 mV.	[40]
1. DC-driven streamer corona. 2. Transient spark discharge	10 mM	(~10 mA) with a 5–20 kHz repetition rate, 10 kV	The pH changed reduced by 4 units.	[44]
Air plasma generator with copper electrodes and quartz dielectric	2 cM	20 kHz, high voltage (not specified)	The pH changed from 6.8 to 2.3, ORP changed from 250 to 540 mV.	[45]
Atmospheric cold plasma jet	7.5 cM	20 kHz, 30 kV	The pH changed from 5.88 to 2.85, ORP changed from 406.1 to 565.40 mV.	[46]

**Table 2:** The effect of PAW on microbial inactivation on fruit and vegetables.

Bacterial Strain and Food	PAW Discharger	Input AC Voltage (kV)	Power (W)	Plasma Inducement Time (min)	Treatment Time (min)	Inactivation Achieved	Ref
Aerobic bacteria on apple slices	Hollow fiber-based cold micro plasma jet	10 kV	-	10	5	2 log_10_ CFU/mL	[42]
Aerobic bacterial counts on mung bean sprouts	Atmospheric pressure plasma jet (APPJ) system based on gliding arc discharge in air	5 kV	750 W	0.5	30	2.3 log_10_ CFU/g	[57]
*S. aureus* on fresh-cut kiwifruit slices	Micro plasma array	-	-	-	30	1.8 log_10_ CFU/g	[58]
*S. aureus* on strawberries	APPJ	18 kV		20	15	2.0 log_10_ CFU/g	[55]
Total bacterial count on baby spinach leaves	Surface barrier discharge (SBD) reactor	11 kV	36 W	20	2	1 log_10_ CFU/g	[59]
*Saccharomyces cerevisiae* CICC 1374 inoculated on grape berries	Plasma jet with electrodes and a dielectric	8.2 kV	-	60	30	0.5 log_10_ CFU/g	[60]

**Table 3:** The effect of PAW on microbial inactivation in non-food matrix.

Bacterial Strain and Model System	PAW Discharger	Input Voltage (kV)	Current, Power, or Frequency	Plasma Inducement Time (mins)	Treatment Time (mins)	Inactivation Achieved (log_10_ CFU/mL)	Ref
*Enterobacter aerogenes* in water	Plasma jet with rotating nozzle	295 V	22.5 kHz	NR	5	1.92 ± 0.70	[43]
*Enterobacter aerogenes* in plasma-activated acidified buffer (PAAB)	Plasma jet with rotating nozzle	295 V	22.5 kHz	NR	5	5.11 ± 0.63	[43]
*Hafnia alvei* in disinfecting PAW solutions (9.9 mL)	Glidarc system	NR	NR	5	30	>5.0	[61]
*Staphylococcus epidermidis* ATCC 12228,	Gliding arc type	NR	NR	5	15	<6	[62]
*Leuconostoc mesenteroides* CNRZ 1468 cells in tryptic soy broth (TSB)	Gliding arc type	NR	NR	5	20	<6	[62]
*H. alvei* CIP 5731 cells in TSB	Gliding arc type	NR	NR	5	20	<6	[62]
*S. putrefaciens* in sterile phosphate-buffered saline (PBS)	Atmospheric cold plasma jet	22 kV	NR	5	30	>7	[46]
*S. aureus* (NCTC-8325) cells in TSB	Corona discharge	10 kV	5 mA 50 mW	30	60	5.52 ± 0.23	[63]
*E. coli* in sterile PBS	Atmospheric cold plasma jet	30 kV	NR	5	60	5.7	[46]

NR = not reported.
